# Assessment of community pharmacists’ knowledge, attitudes, awareness, and practice-related responses regarding oral anticoagulants in the Northern West Bank, Palestine: a cross-sectional study

**DOI:** 10.1186/s40780-026-00607-w

**Published:** 2026-07-11

**Authors:** Hanan M. Alayyad, Saja W. Abu Arqoub, Malak A. Abuobaid, Lina H. Amer, Amer A. Koni, Dania Abuhalima, Samah W. Al-Jabi, Banan M. Aiesh, Sa’ed H. Zyoud

**Affiliations:** 1 Department of Clinical and Community Pharmacy, Faculty of Pharmacy, An-Najah National University, Nablus, 44839 Palestine; 2 Department of Biomedical Sciences and Basic Clinical Skills, Faculty of Medicine and Allied Medical Sciences, An-Najah National University, Nablus, Palestine; 3 Department of Medicine, Faculty of Medicine and Allied Medical Sciences, An-Najah National University, Nablus, Palestine; 4 Department of Infection Prevention and Control, An-Najah National University Hospital, Nablus, 44839 Palestine; 5 Poison Control and Drug Information Center (PCDIC), College of Medicine and Health Sciences, An-Najah National University, Nablus, 44839 Palestine; 6 Clinical Research Center, An-Najah National University Hospital, Nablus, 44839 Palestine

**Keywords:** Direct oral anticoagulants, Warfarin, Community pharmacists, Knowledge, Counselling, Medication safety

## Abstract

**Background:**

Oral anticoagulants, including warfarin and direct oral anticoagulants (DOACs), are widely used for the prevention and treatment of thromboembolic disorders. Community pharmacists are accessible healthcare professionals who may support safe use through counselling on adherence, interactions, adverse effects, and monitoring requirements. This study assessed community pharmacists’ knowledge, attitudes, awareness, and practice-related responses regarding warfarin and DOACs and examined factors associated with knowledge scores.

**Methods:**

This cross-sectional study was conducted among community pharmacists working in the northern West Bank, Palestine, between October 2022 and March 2023. A total of 340 pharmacists were approached, and 329 completed the questionnaire (response rate: 96.8%). Data were collected using a structured 70-item questionnaire covering sociodemographic and professional characteristics, knowledge, awareness and attitudes, and practice-related items. Descriptive statistics were used to summarize participant characteristics and questionnaire responses. Knowledge scores were compared across subgroups using Mann-Whitney U and Kruskal-Wallis tests, and multivariable linear regression was used to examine independent associations with knowledge scores. A p value < 0.05 was considered statistically significant.

**Results:**

The study included 329 pharmacists with a mean age of 33.64 years; 70.5% were women and 80.9% held a bachelor’s degree in pharmacy. Warfarin (Coumadin 5 mg) was available in 87.8% of participating pharmacies. Across knowledge items, 49.1% of responses were correct. Only 48.6% correctly recognized that missing a single DOAC dose could worsen a patient’s condition, and 49.2% selected “do not know” regarding the interaction of apixaban or rivaroxaban with St. John’s wort. For warfarin, 84.8% recognized potential dietary interactions and 84.2% recognized the importance of international normalized ratio (INR) monitoring, whereas only 35.3% identified vitamin K as a reversal option for excessive warfarin anticoagulation or bleeding. In univariable analyses, no statistically significant differences in knowledge scores were observed according to sociodemographic or professional characteristics (all *p* > 0.05). Consistent with these findings, the multivariable model was not statistically significant, and no independent predictors of knowledge score were identified.

**Conclusions:**

Community pharmacists in the northern West Bank showed suboptimal knowledge regarding oral anticoagulants, with important gaps related to DOAC reversal agents, drug and herbal interactions, and missed-dose counselling. No independent predictors of knowledge score were identified, suggesting that educational needs are broadly distributed among pharmacists. Targeted continuing education focused on practical anticoagulant counselling and medication-safety issues may strengthen pharmacists’ contribution to safer oral anticoagulant use.

**Supplementary Information:**

The online version contains supplementary material available at https://doi.org/10.1186/s40780-026-00607-w.

## Background

Direct oral anticoagulants (DOACs) have become increasingly used alternatives to vitamin K antagonists (VKAs) for selected thromboembolic and cardiovascular indications [1–3]. Their introduction expanded available oral anticoagulation options and reduced reliance on warfarin in several clinical settings [2, 4]. Evidence and guideline-based practice support their use for specific prevention and treatment indications, including venous thromboembolism and stroke prevention in eligible patients with nonvalvular atrial fibrillation [1–3, 5, 6].

Warfarin is a vitamin K antagonist that remains an important oral anticoagulant in the treatment and prevention of thromboembolic disorders [7–10]. It also remains widely used in clinical practice [11]. Its safe use requires careful monitoring because of its narrow therapeutic index, variable dose response, and clinically relevant drug-food and drug-drug interactions [12–15]. Appropriate international normalized ratio (INR) monitoring is therefore central to safe and effective warfarin therapy [16–18]. DOACs include factor Xa inhibitors, such as rivaroxaban, apixaban, and edoxaban, and the direct thrombin inhibitor dabigatran [8, 19, 20]. For eligible indications and patient groups, DOACs are commonly preferred over VKAs in current practice recommendations [21–25]. However, their safe use still requires attention to dosing, renal and hepatic function, interacting medicines, and conditions that may alter absorption, including major gastrointestinal surgery [26].

Because oral anticoagulants are associated with clinically important bleeding and interaction risks, pharmacists require sufficient knowledge to provide safe counselling and identify medication-related problems [8, 14, 26, 27]. Papastergiou et al. emphasized the need to strengthen pharmacists’ confidence and education in anticoagulant care as the use of non-vitamin K antagonist oral anticoagulants increased [28]. Similarly, Tan et al. reported greater pharmacist confidence in providing care related to warfarin than to newer anticoagulants [7]. These findings support the need for continuing education that addresses both established and newer oral anticoagulant therapies.

In Palestine, patients requiring oral anticoagulant therapy are generally diagnosed and initiated on treatment by physicians in hospitals or specialized clinics. Warfarin treatment requires regular INR monitoring, usually performed in hospitals or laboratories. Patients commonly obtain medicines from community pharmacies, where pharmacists may reinforce adherence, advise on missed doses and interactions, identify adverse-effect concerns, and remind patients about monitoring requirements. Community pharmacists may therefore represent an important medication-safety contact for patients receiving oral anticoagulants, particularly where specialized anticoagulation services are limited.

Although pharmacists require current knowledge to support safe anticoagulant use, limited evidence is available on community pharmacists’ knowledge and practice-related competence regarding oral anticoagulants in Palestine. Studies from Jordan, Qatar, and Saudi Arabia have evaluated pharmacists’ knowledge of anticoagulant therapy [14, 16, 27], but healthcare infrastructure, access to monitoring services, patterns of medicine availability, and counselling opportunities may differ from those in Palestine. Assessing local knowledge gaps is important because anticoagulant-related counselling involves issues with direct implications for patient safety, including adherence, missed doses, bleeding, drug and herbal interactions, and warfarin monitoring. Therefore, this study aimed to clarify the extent and pattern of community pharmacists’ knowledge, attitudes, awareness, and practice-related responses regarding warfarin and DOACs in the northern West Bank, Palestine, and to determine whether these knowledge gaps were concentrated within specific demographic or professional subgroups. By generating local evidence on anticoagulant counselling and medication-safety gaps, the study may inform continuing professional education and support safer oral anticoagulant use in routine community pharmacy practice.

## Methods

### Study design and timing

This cross-sectional questionnaire-based study targeted pharmacists working in community pharmacies in the northern West Bank, Palestine. Data were collected between October 2022 and March 2023.

### Study population and setting

The study was conducted in community pharmacies located in the northern West Bank, including Nablus, Jenin, Tulkarm, Salfit, and Qalqilya, as well as their surrounding villages and camps. According to the Palestinian Ministry of Health annual report for 2021, 491 licensed community pharmacies were operating in these areas [29]. Based on an assumed average of three pharmacists per pharmacy, the estimated source population was 1,473 pharmacists. Eligible participants were licensed pharmacists currently working in community pharmacies in the study area. Pharmacy students, pharmacy technicians, and pharmacists not currently practicing in community pharmacies were excluded. Pharmacists were approached in person during working hours and invited to participate voluntarily after verbal informed consent was obtained.

### Sample size and sampling procedure

The minimum sample size was estimated using the Raosoft online sample size calculator, based on an estimated population of 1,473 pharmacists, a 5% margin of error, a 95% confidence level, and a 50% response distribution. The calculated minimum sample size was 305 pharmacists. To allow for possible non-response, the target sample was increased to 340 pharmacists. Data were collected through face-to-face administration of a structured questionnaire using a non-probability convenience sampling approach. Completion required approximately 10–15 min. Although convenience sampling may introduce selection bias, pharmacists from different cities and pharmacy locations were approached to increase diversity within the recruited sample.

### Data collection form

Data were collected using a structured paper-based questionnaire prepared in English (Additional file 1), accompanied by an Arabic translation of all questions to support participant comprehension. The questionnaire was developed from previously published studies assessing pharmacists’ knowledge, attitudes, and practices regarding anticoagulants, with items adapted from instruments used in similar settings [14, 27, 30–33]. Questions concerning DOAC knowledge and awareness were mainly adapted from El-Bardissy et al. [27, 30], whereas warfarin-related items were adapted from Tahaineh et al. [27] and studies evaluating food-drug interactions [32, 33]. Items were adopted or modified according to the local context and the study objectives. In this study, “knowledge” referred to understanding of indications, monitoring, interactions, contraindications, and adverse effects; “awareness” referred to understanding of safety and efficacy considerations in selected clinical situations; and “practice-related responses” referred to counselling and medication-management decisions relevant to anticoagulant dispensing, including administration, interactions, and adherence.

The questionnaire consisted of closed-ended items with three response options: “true”, “false”, and “I do not know”. The final questionnaire included 70 items divided into four main sections.

In the first section, the sociodemographic and professional characteristics of the community pharmacists, including age, sex, years of experience, educational level, pharmacy location, and availability of oral anticoagulant medications in the pharmacy, were collected. In this study, “camp” refers to Palestinian refugee camps, which are residential areas originally established for displaced populations and now function as urban communities. “Village” refers to rural residential areas with smaller populations than cities do.

The second section evaluated pharmacists’ knowledge regarding warfarin and direct oral anticoagulants (DOACs), including indications, mechanisms of action, administration, monitoring, contraindications, adverse effects, and drug and food interactions. Each correct answer in the knowledge section was assigned one point, whereas incorrect and “I do not know” responses were assigned zero points. The total knowledge score was calculated by summing the correct responses for each participant. All knowledge items were weighted equally. Higher scores indicated better knowledge regarding anticoagulant therapy.

The third section assessed awareness and attitudes regarding oral anticoagulants, including items related to safety, efficacy, and clinical decision-making. In this study, awareness represented understanding of safety and efficacy considerations in selected situations, whereas attitudes represented pharmacists’ perceptions and intended recommendations related to anticoagulant use.

The fourth section evaluated practice-related responses concerning DOAC dispensing and patient counselling. The items assessed counselling and medication-management decisions relevant to patients receiving DOACs. Two items addressed clinically relevant drug interactions, including rivaroxaban-phenytoin and dabigatran-cyclosporine interactions, because identifying potential interactions is a routine responsibility during dispensing. The remaining items addressed administration and patient counselling. These items should therefore be interpreted as practice-related knowledge or intended counselling responses rather than direct observation of actual practice.

### Validity and reliability

The questionnaire underwent face and content validity assessment by a panel of four experts, including pharmacists and a linguist, using the Arabic version presented to participants. The panel assessed organization, clinical terminology, completeness, appropriateness, logical sequence, and statement accuracy, and revisions were made when needed. A pilot test involving 15 pharmacists was then conducted to assess readability and initial reliability; pilot responses were excluded from the final analysis. Cronbach’s alpha values were 0.784 for the knowledge domain, 0.723 for the attitudes and awareness domain, and 0.814 for the practice-related domain. Values greater than 0.70 were considered indicative of acceptable internal consistency for these domains.

### Statistical analysis

Data were analysed using IBM SPSS Statistics version 26. Frequencies and percentages were used to summarize categorical variables. Normally distributed continuous variables were summarized using means and standard deviations (SDs), whereas non-normally distributed variables were summarized using medians and interquartile ranges (Q1-Q3). Age was categorized as < = 40 years and > 40 years for subgroup comparisons and to maintain adequate group sizes. Because knowledge scores were not normally distributed according to the Kolmogorov-Smirnov test and visual inspection of histograms, Mann-Whitney U and Kruskal-Wallis tests were used for univariable comparisons. A p value < 0.05 was considered statistically significant. All variables in univariable analyses were entered into a multivariable linear regression model to examine independent associations with knowledge scores.

### Ethical considerations

The study was approved by the Institutional Review Board of An-Najah National University (IRB reference number: Ph.D. Sep. 2022/31). The study objectives were explained to eligible pharmacists, and verbal informed consent was obtained before participation. Verbal consent was approved by the IRB because participation involved completion of a questionnaire without intervention and confidentiality was maintained. All methods were conducted in accordance with relevant ethical guidelines and regulations.

## Results

### Sociodemographic characteristics of the study sample

Of the 340 pharmacists approached, 329 participated, yielding a response rate of 96.8%. The mean age was 33.64 ± 11.08 years. Of the participants, 232 (70.5%) were women and 97 (29.5%) were men. Most held a bachelor’s degree in pharmacy (80.9%), 11.9% held a Doctor of Pharmacy degree, and 7.3% held a master’s degree. Approximately half of the pharmacists had ≤ 5 years of experience, and 61.1% reported a monthly income of 2,000–5,000 NIS. More than half (53.2%) resided in cities. Details are presented in Table 1. Warfarin (Coumadin 5 mg) was the most frequently available oral anticoagulant product in participating pharmacies (87.8%). Among DOAC products, Rivarox and Queset were more commonly available than Pradaxa (Additional File 2: Supplementary Table S1).

**Table 1:** Sociodemographic characteristics of community pharmacists

Variable	Frequency *N* = 329	Percentage (%)
Age (years) ≤40 > 40	249 80	75.7 24.3
Gender Male Female	97 232	29.5 70.5
**Degree of education** Bachelor’s degree in pharmacy Pharm D Master	266 39 24	80.9 11.9 7.3
**Duration of experience** (years) ≤ 5 > 5	164 165	49.8 50.2
**District** Nablus Tulkarm Qalqilya Jenin Salfit	114 61 27 92 35	34.7 18.5 8.2 28.0 10.6
**Pharmacy location** Camp Village City	24 107 198	7.3 32.5 60.2
**Pharmacists residence** Camp Village City	12 142 175	3.6 43.2 53.2
Pharmacists income* ** <** 2000 2000–5000 5000–10,000 > 10,000	93 201 32 3	28.3 61.1 9.7 0.9
**University of Graduation** Local University International University	257 72	78.1 21.9
**Pharmacist’s position** Employer Employee	103 226	31.3 68.7

*Income categories are presented in New Israeli Shekels (NIS) per month

### Knowledge of community pharmacists regarding warfarin and DOACs

Knowledge was evaluated across items addressing indications, mechanisms of action, administration, monitoring, food-drug and drug-drug interactions, contraindications, precautions, and adverse effects. Across all knowledge items, 49.1% of responses were correct, 30.7% were incorrect, and 20.2% were “do not know” responses (Table 2).

**Table 2:** Knowledge of community pharmacists about warfarin and DOACs

Sections	Questions #	Answer	Correct answer		Incorrect answer		Don’t know	
			*N*	(%)	*N*	(%)	*N*	(%)
Participants’ knowledge of indications, mechanisms of action, and administration-related issues of DOACs.	Is it acceptable to take these medications at different times as long as they are taken on the day needed?	No	260	(79.0)	63	(19.1)	6	(1.8)
	Is it acceptable to double the next dose of these medications if they were missed?	No	309	(93.9)	10	(3.0)	10	(3.0)
	Is it possible that skipping one dose of these medications could worsen the patient’s condition?	Yes	160	(48.6)	128	(38.9)	41	(12.5)
	Is it appropriate to stop taking these medications once patients feel better?	No	310	(94.2)	16	(4.9)	3	(0.9)
	Is it safe to take anti-inflammatory medications like ibuprofen while patients take these medications?	No	230	(69.9)	66	(20.1)	33	(10.0)
Participant’s knowledge of drug interactions, contraindications, precautions, adverse effects, and other related issues of DOACs	Is it safe to take vitamins and herbal medicines with these medications?	No	229	(69.6)	73	(22.2)	27	(8.2)
	Will drinking too much alcohol increase the risk of side effects with these medications?	Yes	288	(87.5)	24	(7.3)	17	(5.2)
	Is it necessary to inform a surgeon, dentist, or other healthcare professional that patients are taking these medications before undergoing surgery or a procedure?	Yes	319	(97.0)	6	(1.8)	4	(1.2)
	Is it important that all healthcare professionals know that a patient is taking these medications?	Yes	310	(94.2)	11	(3.3)	8	(2.4)
	Is bleeding the most important side effect of these drugs?	Yes	306	(93.0)	14	(4.3)	9	(2.7)
Community pharmacists’ knowledge about warfarin	The INR value is the most important monitoring parameter for warfarin?	Yes	277	(84.2)	6	(1.8)	46	(14.0)
	Is the INR target range (4–5.5)?	No	171	(52.0)	62	(18.8)	96	(29.2)
	Is it possible that subtherapeutic INR is harmful for health?	Yes	210	(63.8)	47	(14.3)	72	(21.9)
	Is it possible that diet affects warfarin therapy?	Yes	279	(84.8)	27	(8.2)	23	(7.0)
	Does ciprofloxacin interact with warfarin?	Yes	250	(76.0)	27	(8.2)	52	(15.8)
	Does diclofenac interact with warfarin?	Yes	226	(68.7)	54	(16.4)	49	(14.9)
	Is vitamin K used to reverse the effect of warfarin in cases of excessive anticoagulation or bleeding?	Yes	116	(35.3)	191	(58.1)	22	(6.7)
Knowledge of community pharmacists about the interactions of warfarin and DOACs with foods	Evaluate the degree of interaction between warfarin and broccoli.	moderate	87	(26.4)	153	(46.5)	89	(27.1)
	Evaluate the degree of interaction between warfarin and caffeine or caffeinated drinks.	minor	66	(20.1)	211	(64.2)	52	(15.8)
	Evaluate the degree of interaction between warfarin and cannabis (*Cannabis sativa*).	Moderate	44	(13.4)	190	(57.7)	95	(28.9)
	Evaluate the degree of interaction between warfarin and chamomile (*Matricaria recutita*).	Major	49	(14.9)	187	(56.9)	93	(28.3)
	Evaluate the degree of interaction between warfarin and Ginkgo (*Ginkgo biloba*).	Major	117	(35.6)	116	(35.2)	96	(29.2)
	Evaluate the degree of interaction between warfarin and ginger (*Zingiber officinale*).	Moderate	90	(27.4)	170	(51.6)	69	(21.0)
	Evaluate the degree of interaction between warfarin and fenugreek (*Trigonella foenum-graecum*).	Moderate	88	(26.7)	117	(35.6)	124	(37.7)
	Evaluate the degree of interaction between warfarin and garlic (*Allium sativum*).	Minor	44	(13.4)	226	(68.7)	59	(17.9)
	Evaluate the degree of interaction between warfarin and grapefruit (Citrus paradisi).	Major	193	(58.7)	85	(25.8)	51	(15.5)
	Evaluate the degree of interaction between warfarin and green tea (*Camellia sinensis*).	Moderate	77	(23.4)	167	(50.7)	85	(25.8)
	Evaluate the degree of interaction between warfarin and licorice (*Glycyrrhiza glabra*).	Moderate	86	(26.1)	141	(42.9)	102	(31.0)
	Evaluate the degree of interaction between warfarin and peppermint tea.	Moderate	64	(19.5)	157	(47.7)	108	(32.8)
	Evaluate the degree of interaction between warfarin and pomegranate juice.	Major	51	(15.5)	154	(46.8)	124	(37.7)
	Evaluate the degree of interaction between warfarin and spinach.	Moderate	66	(20.1)	199	(60.5)	64	(19.5)
	Evaluate the degree of interaction between warfarin and tobacco smoking.	Moderate	101	(30.7)	155	(47.2)	73	(22.2)
	Evaluate the degree of interaction between warfarin and St. John’s wort (Hypericum perforatum).	Major	97	(29.5)	84	(25.5)	148	(45.0)
	Evaluate the degree of interaction between dabigatran and cinnamon (Cinnamomum verum)/ginger (Zingiber officinale).	Major	107	(32.5)	105	(31.9)	117	(35.6)
	Evaluate the degree of interaction between rivaroxaban and saffron (*Crocus sativus*).	Major	46	(14.0)	124	(37.7)	159	(48.3)
	Evaluate the degree of interaction between apixaban or rivaroxaban and St. John’s wort (Hypericum perforatum).	Major	92	(28.0)	75	(22.8)	162	(49.2)
	**Mean**			**49.1**		**30.7**		**20.2**

# These questions were adapted from previous studies [14, 27, 32, 33]

DOACs: Direct oral anticoagulants. INR: International normalized ratio

For DOAC indications, mechanisms, and administration-related items, only 48.6% correctly recognized that missing a single dose could worsen a patient’s condition, whereas 94.2% correctly indicated that treatment should not be stopped simply because a patient feels better. Regarding interactions, contraindications, and precautions, 97.0% correctly identified the need for patients to inform surgeons, dentists, or other healthcare professionals about DOAC treatment before procedures, and 69.6% recognized that vitamins and herbal medicines may not be safe during anticoagulant therapy. For warfarin-related items, 84.8% recognized potential dietary interactions and 84.2% recognized the importance of INR monitoring. However, only 35.3% correctly identified vitamin K as a reversal option for excessive warfarin anticoagulation or bleeding.

For food and herbal interaction items, correct-response percentages varied widely. The highest reported correct-response percentage in this subsection was for the grapefruit-warfarin item (58.7%), whereas only 13.4% correctly answered the items relating to warfarin with cannabis and garlic. Nearly half of the pharmacists (49.2%) selected “do not know” for the interaction of apixaban or rivaroxaban with St. John’s wort. Further details are presented in Table 2.

### Attitudes and awareness of pharmacists regarding warfarin and DOACs

Attitudes and awareness were assessed using items related to safety and efficacy. Across these items, 44.7% of responses were correct, 26.0% were incorrect, and 29.3% were “do not know” responses (Table 3).

**Table 3:** Attitudes and awareness of pharmacists toward warfarin and DOACs

Sections	Questions	Answer	Correct answer		Incorrect answer		Don’t know	
			*N*	(%)	*N*	(%)	*N*	(%)
Pharmacists’ Attitude and Awareness toward DOAC and warfarin *safety*.	Can all DOACs be used safely in patients with renal impairment without assessing renal function or considering dose adjustment or avoidance?	No	199	(60.5)	74	(22.5)	56	(17.0)
	Can DOACs be used safely in all patients with moderate to severe hepatic impairment?	No	218	(66.3)	57	(17.3)	54	(16.4)
	Are DOACs used safely during pregnancy and lactation?	No	233	(70.8)	74	(22.5)	22	(6.7)
	Do DOACs interact with leafy green vegetables?	No	82	(24.9)	207	(62.9)	40	(12.2)
	DOACs are associated with fewer major bleeding events than warfarin?	Yes	225	(68.4)	43	(13.1)	61	(18.5)
	DOACs are associated with a lower risk of intracranial hemorrhage than warfarin?	Yes	216	(65.7)	35	(10.6)	78	(23.7)
	All DOACs are associated with a lower risk of gastrointestinal bleeding than warfarin.	No	70	(21.3)	190	(57.8)	69	(21.0)
	Would you recommend DOACs to patients with mechanical valve replacements?	No	62	(18.8)	160	(48.6)	107	(32.5)
	Idarucizumab is the antidote for dabigatran?	Yes	139	(42.2)	31	(9.4)	159	(48.3)
	Andexanet alfa is the antidote for rivaroxaban?	Yes	127	(38.6)	38	(11.6)	164	(49.8)
B 1–7 Attitude and awareness toward DOAC efficacy.	Can the effect of DOACs be easily monitored?	Yes	160	)48.6)	125	(38)	44	(13.4)
	The recommended daily dose of rivaroxaban for DVT and PE treatment is 20 mg beginning on day 1.	No	136	(41.3)	99	(30.1)	94	(28.6)
	DOACs are currently approved for the prevention of VTE in patients with hip or knee replacements?	Yes	158	(48)	33	(10)	138	(41.9)
	DOACs are currently approved for DVT and PE treatment?	Yes	216	(65.7)	29	(8.8)	84	(25.5)
	DOACs are currently approved for stroke prevention in patients with nonvalvular atrial fibrillation?	Yes	150	(45.6)	30	(9.1)	149	(45.3)
	UFH or LMWH are recommended with the initiation of rivaroxaban for the treatment of DVT and PE?	No	57	(17.3)	111	(33.7)	161	(48.9)
	Would you recommend DOACs to patients with mechanical valve replacement?	No	54	(16.3)	116	(35.3)	159	(48.3)
	**Mean**			**44.7**		**26**		**29.3**

# These questions were adapted from previous studies [14, 30, 31]

DOACs: Direct oral anticoagulants. UFH: Unfractionated heparin. LMWH: Low molecular weight heparin.PE: Pulmonary embolism. VTE: Venous thromboembolism. DVT: Deep vein thrombosis. GIT: gastrointestinal tract.

Regarding safety-related items, 48.3% and 49.8% of participants selected “do not know” for the antidotes of dabigatran and rivaroxaban, respectively. In addition, 70.8% correctly identified that DOACs were not considered safe during pregnancy and lactation in the questionnaire item. Regarding bleeding-related items, 68.4% correctly identified that DOACs are associated with fewer major bleeding events than warfarin, and 65.7% recognized their lower risk of intracranial haemorrhage.

Regarding efficacy-related items, 65.7% correctly identified that DOACs are approved for the treatment of deep vein thrombosis and pulmonary embolism. Only 17.3% correctly identified that unfractionated heparin or low-molecular-weight heparin is not routinely required when initiating rivaroxaban for these indications; 82.7% answered incorrectly or selected “do not know”.

### Practice-related responses of community pharmacists regarding DOACs

In the practice-related section, 61.1% correctly identified the clinically relevant interaction between phenytoin and rivaroxaban, and 66.6% correctly indicated that dabigatran capsules should not be chewed, opened, or broken (Table 4).

**Table 4:** Practice-related responses of community pharmacists regarding DOACs

Question	Answer*	Correct answer *N* (%)	Incorrect answer *N* (%)	Don’t know *N* (%)
1. Rivaroxaban for nonvalvular atrial fibrillation should be administered with the evening meal?	Yes	156 (47.4)	49 (14.9)	124 (37.7)
2. Phenytoin interacts significantly with rivaroxaban and should be avoided?	Yes	201 (61.1)	34 (10.3)	94 (28.6)
3. Dabigatran capsules should NOT be chewed, opened, or broken?	Yes	219 (66.6)	26 (7.9)	84 (25.5)
4. Cyclosporine interacts significantly with dabigatran and should be avoided?	Yes	173 (52.6)	20 (6.1)	136 (41.3)

DOACs: Direct oral anticoagulants

### Associations between sociodemographic variables and pharmacist knowledge

Univariable analyses examined differences in knowledge scores according to participant characteristics. No statistically significant differences were observed in knowledge scores across age, gender, duration of experience, graduation university, education level, city of work, pharmacy location, residence, or income (all *p* > 0.05). Although variations in mean ranks were observed across some categories, these differences did not reach statistical significance (Table 5).

**Table 5:** Associations between sociodemographic variables and pharmacist knowledge

Variable	Frequency (%)	Knowledge score median [Q1–Q3]	Mean rank	*p*-value*
**Age (years)**				0.480ᵇ
≤ 40	249 (75.7)	12 [11–15]	167.09	
> 40	80 (24.3)	12 [11–15]	158.50	
**Gender**				0.944ᵇ
Male	97 (29.5)	12 [11–15]	164.43	
Female	232 (70.5)	12 [11–15]	165.24	
**Duration of experience**				0.971ᵇ
≤ 5 years	164 (49.8)	12 [11–15]	164.81	
> 5 years	165 (50.2)	12 [11–15]	165.19	
**Graduation university**				0.371ᵇ
Local	257 (78.1)	12 [11–15]	162.53	
International	72 (21.9)	12 [11–15]	173.83	
**Education level**				0.084ᵃ
Bachelor	266 (80.9)	12 [11–15]	159.49	
PharmD	39 (11.9)	12 [11–15]	192.87	
Master	24 (7.3)	12 [11–15]	180.77	
**City of work**				0.098ᵃ
Nablus	114 (34.7)	12 [11–15]	161.57	
Tulkarm	61 (18.5)	12 [11–15]	161.98	
Qalqilya	27 (8.2)	12 [11–15]	130.78	
Jenin	92 (28.0)	12 [11–15]	184.35	
Salfit	35 (10.6)	12 [11–15]	156.99	
**Pharmacy location**				0.293ᵃ
Camp	24 (7.3)	12 [11–15]	137.08	
Village	107 (32.5)	12 [11–15]	163.83	
City	198 (60.2)	12 [11–15]	169.02	
**Pharmacists’ residence**				0.586ᵃ
Camp	12 (3.6)	12 [11–15]	180.63	
Village	142 (43.2)	12 [11–15]	159.37	
City	175 (53.2)	12 [11–15]	168.50	
**Pharmacists income**				0.163ᵃ
< 2000	93 (28.3)	12 [11–15]	154.26	
2000–5000	201 (61.1)	12 [11–15]	167.66	
5000–10,000	32 (9.7)	12 [11–15]	186.88	
> 10,000	3 (0.9)	12 [11–15]	86.50	

* The value denotes statistical significance

a Kruskal‒Wallis test

b Mann‒Whitney U test

### Multivariable linear regression analysis

A multivariable linear regression model was fitted to examine independent associations with knowledge score (Table 6). The overall model was not statistically significant (F = 1.264, *p* = 0.256) and explained a small proportion of the variance in knowledge scores (R² = 0.034). After adjustment for sociodemographic and professional variables, none of the included predictors were significantly associated with knowledge score at the 0.05 level. Graduation university (B = 1.103, *p* = 0.088), education level (B = 0.578, *p* = 0.131), and income (B = 0.588, *p* = 0.131) showed borderline associations, but these did not reach statistical significance.

**Table 6:** Multivariable linear regression analysis of factors associated with knowledge score

Variable	B	Std. Error	Beta	95% CI	*p*-value
Age group	-0.804	0.678	-0.086	-2.139–0.530	0.237
Gender	0.598	0.588	0.068	-0.559–1.755	0.310
Duration of experience (2-category)	0.270	0.557	0.034	-0.827–1.366	0.629
Graduation university	1.103	0.644	0.114	-0.164–2.370	0.088
Education level	0.578	0.381	0.084	-0.173–1.328	0.131
City of work	0.050	0.156	0.018	-0.256–0.356	0.747
Pharmacy location	0.493	0.406	0.077	-0.306–1.292	0.226
Pharmacists’ residence	-0.097	0.453	-0.014	-0.988–0.794	0.830
Income	0.588	0.388	0.092	-0.176–1.352	0.131

## Discussion

This study assessed community pharmacists’ knowledge, attitudes, awareness, and practice-related responses regarding oral anticoagulants in the northern West Bank, Palestine. Across knowledge items, approximately half of responses were correct, with important gaps relating to DOAC reversal agents, drug and herbal interactions, and missed-dose counselling. Although some variation in mean ranks was observed across participant subgroups, none of the sociodemographic or professional characteristics showed a statistically significant association with knowledge scores in univariable analyses. Therefore, demographic differences observed in univariable analyses should not be interpreted as independent determinants of anticoagulant knowledge. The results instead support the value of broadly accessible continuing education that focuses on DOAC indications, reversal agents, interactions, INR-related counselling, and management of missed doses [27].

These findings are clinically relevant because community pharmacists may be a readily accessible source of medicine-related counselling for patients receiving oral anticoagulants. Knowledge gaps concerning drug or herbal interactions, reversal options, and missed doses may reduce the quality of counselling provided at the point of dispensing. Evidence from Palestine also supports the importance of patient-centred counselling in anticoagulant therapy. A previous study among Palestinian patients receiving anticoagulants reported that treatment satisfaction was positively associated with self-efficacy, and that patients receiving newer oral anticoagulants had higher self-efficacy and perceived treatment benefit scores than those receiving vitamin K antagonists. These findings highlight the potential role of community pharmacists in improving patients’ understanding of anticoagulant therapy, supporting adherence, addressing treatment concerns, and promoting safer medication use [34]. To the best of our knowledge, this is the first study to evaluate community pharmacists’ knowledge, attitudes, awareness, and practice-related responses regarding warfarin and DOACs in Palestine.

The observed variation in knowledge scores across cities may reflect differences in exposure to oral anticoagulant prescriptions, access to hospitals or specialized clinics, and opportunities for continuing education. However, this variation was not statistically significant and should be interpreted cautiously.

A study from Qatar evaluated pharmacists’ knowledge and attitudes regarding DOACs and reported that knowledge gaps were particularly evident in efficacy-related items [14]. In the present study, neither age nor years of experience was significantly associated with knowledge scores in the univariable analyses, and no demographic or professional variable remained significant in the multivariable model. This consistency between the univariable and adjusted findings resolves the possibility that knowledge gaps were confined to a specific pharmacist subgroup. Instead, the findings indicate that gaps in anticoagulant knowledge were broadly distributed across the participating community pharmacists.

Response patterns suggested greater familiarity with several warfarin-related issues, such as diet interactions and INR monitoring, than with selected DOAC-related issues, including reversal agents and interactions. This may be explained by the longer history of warfarin use and the greater exposure of pharmacists to its monitoring and counselling requirements. In contrast, DOAC-specific counselling may require updated training on dosing, indications, reversal agents, and interacting medicines.

Medication availability may also influence familiarity in routine community pharmacy practice. Coumadin, Rivarox, and Queset were more commonly available in participating pharmacies than Pradaxa products, potentially increasing exposure to some agents while limiting experience with others.

The correct-response percentage for warfarin-related knowledge items was higher than the overall knowledge percentage, particularly for dietary interactions and INR monitoring. Similar knowledge gaps have been reported among community pharmacists in Jordan [27], supporting the relevance of continuing education on oral anticoagulants across regional practice settings.

Previous research in Saudi Arabia also evaluated pharmacists’ knowledge and counselling practices regarding warfarin [16]. In the present study, correct-response percentages for selected INR-related items appeared higher than those reported in some regional studies [16, 27]. Nevertheless, differences in questionnaire content, healthcare systems, medicine availability, and pharmacist training mean that such comparisons should be interpreted cautiously.

The absence of statistically significant differences across demographic and professional characteristics is an important finding. It suggests that suboptimal anticoagulant knowledge was not limited to younger pharmacists, older pharmacists, pharmacists with fewer years of experience, or pharmacists working in particular districts. Educational initiatives should therefore be comprehensive and offered to all community pharmacists rather than targeted only to selected subgroups. Priority topics should be directly linked to the gaps observed in this study, including DOAC reversal agents, clinically relevant drug and herbal interactions, approved indications, administration requirements, counselling on missed doses, and recognition of bleeding risk. Collaboration between professional pharmacy bodies, academic institutions, and health authorities may support practical, case-based continuing education for community pharmacists. Such programmes should be evaluated in future studies to determine whether they improve knowledge, counselling performance, and medication-safety practices.

### Strengths and limitations

This study has several strengths. It recruited a relatively large number of community pharmacists from multiple cities in the northern West Bank and assessed several clinically relevant domains of oral anticoagulant knowledge and counselling. Several limitations should also be considered. First, convenience sampling may have introduced selection bias and limits generalizability beyond the recruited pharmacists. Second, the study was restricted to the northern West Bank; consequently, the results should not be generalized to all community pharmacists in Palestine, including pharmacists in other regions. Third, responses were self-reported and practice-related items reflected intended responses rather than direct observation of counselling behaviour. Fourth, although face and content validity and internal consistency were assessed, further psychometric evaluation, including item discrimination, was not performed. Fifth, differences in the availability of individual anticoagulants across pharmacies may have influenced familiarity with specific agents. Finally, the cross-sectional design does not allow causal interpretation of associations between participant characteristics and knowledge scores, and the small size of some subgroup categories may have limited the ability to detect modest differences between pharmacist groups.

## Conclusions

Community pharmacists in the northern West Bank demonstrated suboptimal knowledge regarding oral anticoagulants, correctly answering approximately half of the knowledge items. Important gaps were identified in areas directly related to patient safety, particularly DOAC reversal agents, drug and herbal interactions, and missed-dose counselling. No sociodemographic or professional characteristic was independently associated with knowledge scores, suggesting that educational needs are broadly distributed among community pharmacists. Targeted continuing education and practical training on oral anticoagulant counselling, interaction screening, bleeding-risk recognition, and medication-safety practices may strengthen the role of community pharmacists in supporting safer anticoagulant use in routine practice.

### Recommendations

Community pharmacists can contribute to safer oral anticoagulant use through patient counselling, medication review, and recognition of potential interactions and adverse effects. Counselling at the point of dispensing may be particularly important for adherence, missed-dose management, and timely referral when bleeding concerns arise.

Continuing education programmes focused on oral anticoagulant therapy, particularly DOACs, should be developed in collaboration with relevant health authorities, professional pharmacy bodies, and pharmacy faculties. Priority content should include indications, administration, clinically important interactions, reversal agents, bleeding-risk counselling, and missed-dose management.

Where feasible, continuing professional development related to high-risk medicines could be incorporated into professional education and licence-renewal frameworks. Such initiatives should be evaluated to determine whether they improve pharmacists’ knowledge and patient-counselling performance.

## Supplementary Information

Below is the link to the electronic supplementary material.

Supplementary Material 1

Supplementary Material 2

## Data Availability

The data collected and analysed for this study are available from the corresponding author upon reasonable request.

## Abbreviations

DOACs: Direct oral anticoagulants
GIT: Gastrointestinal tract
PE: Pulmonary embolism
DVT: Deep vein thrombosis
VTE: Venous thromboembolism
UFH: Unfractionated heparin
LMWH: Low-molecular weight heparin
INR: International normalized ratio
IRB: Institutional Review Board
VKAs: Vitamin K antagonists
NOACs: Non-vitamin K antagonist oral anticoagulants
SD: Standard deviation
IBM: International Business Machines
SPSS: Statistical Package for the Social Sciences
Q1: First quartile
Q3: Third quartile
N: Sample size
