# Supplementary material for: Assessment of community pharmacists’ knowledge, attitudes, awareness, and practice-related responses regarding oral anticoagulants in the Northern West Bank, Palestine: a cross-sectional study
Source: J Pharm Health Care Sci. 2026 Jul 11;12:85. doi: 10.1186/s40780-026-00607-w (PMC13644199; doi:10.1186/s40780-026-00607-w)
Supplement: Supplementary file 1 [file 40780_2026_607_MOESM1_ESM.docx]

**Additional File 2:** **Supplementary Table S1.** Anticoagulants available in participating community pharmacies.

**Supplementary Table S1.** Anticoagulants available in participating community pharmacies.

| **Drugs** | **Available** | **Frequency** | **(%)** |
| --- | --- | --- | --- |
| **COUMADIN 1 mg** | YES | 1. 128 | 2. (38.9) |
| **COUMADIN 2 mg** | YES | 3. 116 | 4. (35.3) |
| **COUMADIN 2.5 mg** | YES | 5. 147 | 6. (44.7) |
| **COUMADIN 5 mg** | YES | 7. 289 | 8. (87.8) |
| **RIVOXAR 2.5 mg** | YES | 9. 189 | 10. 57.4)) |
| **RIVOXAR 10 mg** | YES | 11. 261 | 12. 79.3)) |
| **RIVOXAR 15 mg** | YES | 13. 234 | 14. 71.1)) |
| **RIVOXAR 20 mg** | YES | 15. 193 | 16. 58.7)) |
| **XARELTO 2.5 mg** | YES | 17. 123 | 18. 37.4)) |
| **XARELTO 10 mg** | YES | 19. 165 | 20. 50.2)) |
| **XARELTO 15 mg** | YES | 21. 155 | 22. 47.1)) |
| **XARELTO 20 mg** | YES | 23. 161 | 24. 48.9)) |
| **PRADAXA 150 mg** | YES | 25. 46 | 26. 14.0)) |
| **PRADAXA 110 mg** | YES | 27. 43 | 28. 13.1)) |
| **IXABAN 2.5 mg** | YES | 29. 172 | 30. 52.3)) |
| **IXABAN 5 mg** | YES | 31. 168 | 32. 51.1)) |
| **QUESET 2.5 mg** | YES | 33. 265 | 34. 80.5)) |
| **QUESET 5 mg** | YES | 35. 267 | 36. 81.2)) |
| **ELIQUIS 2.5 mg** | YES | 37. 185 | 38. 56.2)) |
| **ELIQUIS 5 mg** | YES | 39. 211 | 40. 64.1)) |
