# Supplementary material for: Assessment of community pharmacists’ knowledge, attitudes, awareness, and practice-related responses regarding oral anticoagulants in the Northern West Bank, Palestine: a cross-sectional study
Source: J Pharm Health Care Sci. 2026 Jul 11;12:85. doi: 10.1186/s40780-026-00607-w (PMC13644199; doi:10.1186/s40780-026-00607-w)
Supplement: Supplementary file 2 [file 40780_2026_607_MOESM2_ESM.docx]

**Additional File 1:** Study questionnaire. This is the final English version of the questionnaire used to assess knowledge, attitudes, awareness, and practices of community pharmacists regarding oral anticoagulants. **Supplementary Table S1.** Anticoagulants available in participating community pharmacies.

**Section 1: Sociodemographic and Professional Characteristics**

Please select or fill in the appropriate answer.

1. Age (years):-----------
2. Gender:
   ☐ Male
   ☐ Female
3. Highest pharmacy degree obtained:
   ☐ Bachelor of Pharmacy
   ☐ Doctor of Pharmacy (PharmD)
   ☐ Master’s degree
   ☐ PhD
   ☐ Other: __________
4. Years of experience in community pharmacy:…………………………
5. City of work:
   ☐ Nablus
   ☐ Jenin
   ☐ Tulkarm
   ☐ Qalqilya
   ☐ Salfit
   ☐ Other: __________
6. Pharmacy location:
   ☐ City
   ☐ Village
   ☐ Camp
7. Place of residence:
   ☐ City
   ☐ Village
   ☐ Camp
8. Monthly income:
   ☐ < 2000
   ☐ 2000–5000
   ☐ 5000–10000
   ☐ > 10000
9. University of graduation:
   ☐ Local university
   ☐ International university
10. Current position:
    ☐ Pharmacy owner / Employer
    ☐ Pharmacy employee
11. Are oral anticoagulants available in your pharmacy?
    ☐ Yes
    ☐ No

**Section 2: Availability of Oral Anticoagulants in the Pharmacy**

**Please indicate whether the following medicines and strengths are available in your pharmacy.**

| **Medicine** | **Strength** | **Yes** | **No** |
| --- | --- | --- | --- |
| COUMADIN | 1 mg | ☐ | ☐ |
| COUMADIN | 2 mg | ☐ | ☐ |
| COUMADIN | 2.5 mg | ☐ | ☐ |
| COUMADIN | 5 mg | ☐ | ☐ |
| RIVOXAR | 2.5 mg | ☐ | ☐ |
| RIVOXAR | 10 mg | ☐ | ☐ |
| RIVOXAR | 15 mg | ☐ | ☐ |
| RIVOXAR | 20 mg | ☐ | ☐ |
| XARELTO | 2.5 mg | ☐ | ☐ |
| XARELTO | 10 mg | ☐ | ☐ |
| XARELTO | 15 mg | ☐ | ☐ |
| XARELTO | 20 mg | ☐ | ☐ |
| PRADAXA | 150 mg | ☐ | ☐ |
| PRADAXA | 110 mg | ☐ | ☐ |
| IXABAN | 2.5 mg | ☐ | ☐ |
| IXABAN | 5 mg | ☐ | ☐ |
| QUESET | 2.5 mg | ☐ | ☐ |
| QUESET | 5 mg | ☐ | ☐ |
| ELIQUIS | 2.5 mg | ☐ | ☐ |
| ELIQUIS | 5 mg | ☐ | ☐ |

**Section 3: Knowledge about Warfarin and DOACs**

Please answer the following questions by selecting:
**True / False / I do not know**

**Indications, Administration, and Monitoring**

1. Is it acceptable to take these medications at different times as long as they are taken on the day needed?
2. Is it acceptable to double the next dose of these medications if they were missed?
3. Is it possible that skipping one dose of these medications could worsen the patient’s condition?
4. Is it appropriate to stop taking these medications once patients feel better?
5. Is it safe to take anti-inflammatory medications like ibuprofen while patients take these medications?
6. Is it safe to take vitamins and herbal medicines with these medications?
7. Will drinking too much alcohol increase the risk of side effects with these medications?
8. Is it necessary to inform a surgeon, dentist, or other healthcare professional that patients are taking these medications before undergoing surgery or a procedure?
9. Is it important that all healthcare professionals know that a patient is taking these medications?
10. Is bleeding the most important side effect of these drugs?

**Warfarin Knowledge**

11. The INR value is the most important monitoring parameter for warfarin.
12. Is the INR target range (4–5.5)?
13. Is it possible that subtherapeutic INR is harmful for health?
14. Is it possible that diet affects warfarin therapy?
15. Does ciprofloxacin interact with warfarin?
16. Does diclofenac interact with warfarin?
17. Is vitamin K used to reverse the effect of warfarin in cases of excessive anticoagulation or bleeding?

**Food and Herbal Interactions**

Indicate the degree of interaction (Major / Moderate / Minor / No interaction / I do not know):

18. Evaluate the degree of interaction between warfarin and broccoli.
19. Evaluate the degree of interaction between warfarin and caffeine or caffeinated drinks.
20. Evaluate the degree of interaction between warfarin and cannabis (*Cannabis sativa*).
21. Evaluate the degree of interaction between warfarin and chamomile (*Matricaria recutita*).
22. Evaluate the degree of interaction between warfarin and Ginkgo (*Ginkgo biloba*).
23. Evaluate the degree of interaction between warfarin and ginger (*Zingiber officinale*).
24. Evaluate the degree of interaction between warfarin and fenugreek (*Trigonella foenum-graecum*).
25. Evaluate the degree of interaction between warfarin and garlic (*Allium sativum*).
26. Evaluate the degree of interaction between warfarin and grapefruit (*Citrus paradisi*).
27. Evaluate the degree of interaction between warfarin and green tea (*Camellia sinensis*).
28. Evaluate the degree of interaction between warfarin and licorice (*Glycyrrhiza glabra*).
29. Evaluate the degree of interaction between warfarin and peppermint tea.
30. Evaluate the degree of interaction between warfarin and pomegranate juice.
31. Evaluate the degree of interaction between warfarin and spinach.
32. Evaluate the degree of interaction between warfarin and tobacco smoking.
33. Evaluate the degree of interaction between warfarin and St. John’s wort (*Hypericum perforatum*).
34. Evaluate the degree of interaction between dabigatran and cinnamon (*Cinnamomum verum*)/ginger (*Zingiber officinale*).
35. Evaluate the degree of interaction between rivaroxaban and saffron (*Crocus sativus*).
36. Evaluate the degree of interaction between apixaban or rivaroxaban and St. John’s wort (*Hypericum perforatum)*

**Section 4: Attitudes and Awareness toward Warfarin and DOACs**

Answer: **True / False / I do not know**

**Safety**

1. Can all DOACs be used safely in patients with renal impairment without assessing renal function or considering dose adjustment or avoidance?
2. Can DOACs be used safely in all patients with moderate to severe hepatic impairment?
3. Are DOACs used safely during pregnancy and lactation?
4. Do DOACs interact with leafy green vegetables?
5. DOACs are associated with fewer major bleeding events than warfarin?
6. DOACs are associated with a lower risk of intracranial hemorrhage than warfarin?
7. All DOACs are associated with a lower risk of gastrointestinal bleeding than warfarin.
8. Would you recommend DOACs to patients with mechanical valve replacements?
9. Idarucizumab is the antidote for dabigatran?
10. Andexanet alfa is the antidote for rivaroxaban?

**Efficacy**

11. Can the effect of DOACs be easily monitored?
12. The recommended daily dose of rivaroxaban for DVT and PE treatment is 20 mg beginning on day 1.
13. DOACs are currently approved for the prevention of VTE in patients with hip or knee replacements?
14. DOACs are currently approved for DVT and PE treatment?
15. DOACs are currently approved for stroke prevention in patients with nonvalvular atrial fibrillation?
16. UFH or LMWH are recommended with the initiation of rivaroxaban for the treatment of DVT and PE?
17. Would you recommend DOACs to patients with mechanical valve replacement?

**Section 5: Practices and Roles of Community Pharmacists**

Answer: **Yes / No / I do not know**

1. Rivaroxaban for atrial fibrillation should be taken with the evening meal.
2. Phenytoin interacts with rivaroxaban and should be avoided.
3. Dabigatran capsules should not be opened, chewed, or crushed.
4. Cyclosporine interacts with dabigatran and should be avoided.
5. Do you counsel patients on anticoagulant adherence?
6. Do you counsel patients on missed doses?
7. Do you counsel patients on bleeding risk?
8. Do you check for drug interactions before dispensing anticoagulants?
9. Do you advise patients to inform doctors/dentists before procedures?
10. Do you provide written information or brochures to patients using anticoagulants?

**Supplementary Table S1.** Anticoagulants available in participating community pharmacies.

| **Drugs** | **Available** | **Frequency** | **(%)** |
| --- | --- | --- | --- |
| **COUMADIN 1 mg** | YES | 1. 128 | 2. (38.9) |
| **COUMADIN 2 mg** | YES | 3. 116 | 4. (35.3) |
| **COUMADIN 2.5 mg** | YES | 5. 147 | 6. (44.7) |
| **COUMADIN 5 mg** | YES | 7. 289 | 8. (87.8) |
| **RIVOXAR 2.5 mg** | YES | 9. 189 | 10. 57.4)) |
| **RIVOXAR 10 mg** | YES | 11. 261 | 12. 79.3)) |
| **RIVOXAR 15 mg** | YES | 13. 234 | 14. 71.1)) |
| **RIVOXAR 20 mg** | YES | 15. 193 | 16. 58.7)) |
| **XARELTO 2.5 mg** | YES | 17. 123 | 18. 37.4)) |
| **XARELTO 10 mg** | YES | 19. 165 | 20. 50.2)) |
| **XARELTO 15 mg** | YES | 21. 155 | 22. 47.1)) |
| **XARELTO 20 mg** | YES | 23. 161 | 24. 48.9)) |
| **PRADAXA 150 mg** | YES | 25. 46 | 26. 14.0)) |
| **PRADAXA 110 mg** | YES | 27. 43 | 28. 13.1)) |
| **IXABAN 2.5 mg** | YES | 29. 172 | 30. 52.3)) |
| **IXABAN 5 mg** | YES | 31. 168 | 32. 51.1)) |
| **QUESET 2.5 mg** | YES | 33. 265 | 34. 80.5)) |
| **QUESET 5 mg** | YES | 35. 267 | 36. 81.2)) |
| **ELIQUIS 2.5 mg** | YES | 37. 185 | 38. 56.2)) |
| **ELIQUIS 5 mg** | YES | 39. 211 | 40. 64.1)) |
